# Precision and effort in robot-assisted placement of pedicle screws compared to standard surgical navigation

**DOI:** 10.1038/s41598-024-77892-8

**Published:** 2024-11-06

**Authors:** Julia Katharina Sippel, Johannes Groh, Lars Bräuer, Mario Perl, Holger Stadthalter

**Affiliations:** 1 Faculty of Medicine, Department of Orthopaedic and Trauma Surgery, Friedrich-Alexander-Universität Erlangen-Nürnberg, Erlangen, Germany; 2 Faculty of Medicine, Institute for Functional and Clinical Anatomy, Friedrich-Alexander-Universität Erlangen-Nürnberg, Erlangen, Germany

**Keywords:** Bone, Medical research

## Abstract

Aim was to compare image-guided navigation with a robot-assisted solution for performing MISS regarding precision, required time and subjective aspects. 90 pedicles were instrumented on two torsos, half with navigation, half robot-assisted. Precision analysis between both solutions didn’t show a significant difference. Time measurement showed a significantly longer duration per wire for the robot-arm on the first torso and a not significant longer duration on the second torso, where a significant reduction in the mean duration was shown. There was no significant difference in the subjective impressions comparing navigation and robot except the possibility to change the procedure. Precision of both methods is suitable for clinical use. A time advantage using the robot-arm couldn’t be demonstrated in the present study. A significant learning curve was shown, so a reduction in the longer duration on the robot can be expected. Further studies in clinical use are necessary.

## Introduction

Medicine is constantly evolving. This especially applies to spinal surgery, that has become ever less invasive, bringing the well-known benefits of reduced approaches, blood loss, soft tissue damage, and thus reduced hospitalization time^1^. As advantageous minimal invasive spine surgery (MISS) has proven for the patient, technically there are challenges in orientation and assurance of correct implant placement. Several technical adjuncts have been developed to address these shortcomings, most noticeably improvements in intraoperative imaging, especially 3D-imaging. During the last decade, optical navigation has additionally become increasingly established to be used in combination with 3D-imaging. This has been shown to increase precision and reduce intraoperative radiation exposure for the patient and especially the surgeon^2,3^. A time advantage could also be shown for optical navigation, especially with multi-level constructs^4^.

Robotic systems have also become commercially available to be used in combination with navigation. Although the first robot for spinal surgery was launched already in 2004^5^, it has only been recently that these systems became feasible for routine use. Various design concepts are currently commercially available, including a compact design, comparable to a conventional articulated arm, which can be mounted on a standard operating table in a space- and weight-saving manner.

In almost all procedures on the spine, the positioning of the pedicle screws is crucial. Rates of malposition vary in the literature and depend on the type of procedure (scoliosis surgery, trauma surgery, degenerative surgery) and range from about 4%^6,8^ to 42%^8^. Misalignment mainly causes nervous complications; the rates range here up to 15%^9^.

The aim of the current study was to compare a clinical standard setting of image-guided navigation (Curve, Brainlab AG, Munich) with a robot-assisted solution (Cirq, Brainlab AG, Munich) for performing minimally invasive percutaneous pedicle screw placement in all levels of the spine in regard of precision and required time. Although there are some clinical reports regarding this robotic system available, a structured evaluation in comparison to the gold standard in navigated procedures has not been published to our knowledge.

The experimental study was designed as a non-inferiority trial.

## Materials and methods

The study was carried out in cooperation with the Institute for Functional and Clinical Anatomy, Friedrich-Alexander-University Erlangen-Nuremberg, on two human specimens. The written informed consent for the use for scientific purposes was given by the individuals during their lifetime in accordance with the standards of the Anatomical Institute. The study was approved by the Institute for Functional and Clinical Anatomy, Friedrich-Alexander-University Erlangen-Nuremberg as well as the ethics committee of the Friedrich-Alexander-University Erlangen-Nuremberg.

All experiments were conducted in accordance with the guidelines and regulations of the ethics committee, the anatomical institute and other applicable ethical principles.

The study was conducted on two fresh-frozen torsos. The experimental setup was the same in each case. Four surgeons of different clinical experience in complex spinal surgery (1–20 years), pedicles of vertebrae C4-S1 and the SI-joint were instrumented using 1.9 mm K-wires (*n* = 90).

K-wires were used instead of screws, as the main aim was to compare the initial implant placement. Both methods were performed in the same way, so it was assumed that the implantation of screws would not provide any additional information. In contrast, it was assumed that due to the small diameter of the wires, deviations in position and angulation could be detected more accurately.

**Fig. 1:**
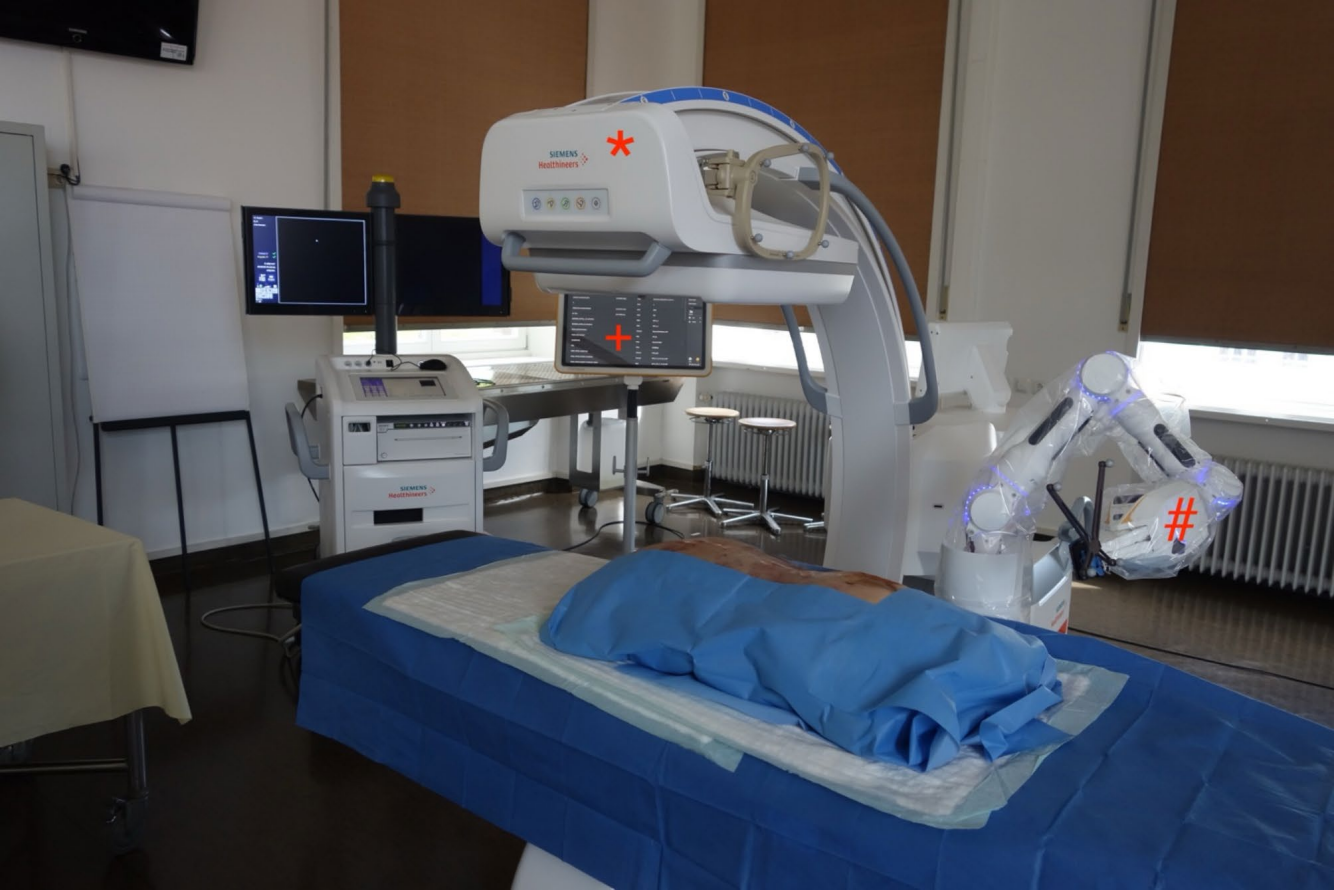
Experimental set-up: *: 3D-C-arm, #: robotic arm, +: navigation display cart.

One side was instrumented with conventional navigation (Curve, Brainlab AG, Munich), the opposite side by the same surgeon with the robot-assisted solution (Cirq, Brainlab AG, Munich). The specimens were positioned on a mobile radiolucent carbon-fiber surgical table in prone position. The navigation camera, the patient reference, the C-arm reference and the display monitor were the same for both procedures. The experimental setup is shown in Fig. 1.

The number of vertebrae to be instrumented was determined by the number of vertebral bodies imaged per scan (4–7 vertebral bodies). The assignment of the surgeon to the corresponding section was randomized. All test subjects performed the procedures on both torsos.

First, a 3D scan of the target section was performed with a flat detector C-arm (Cios Spin, Siemens Healthineers AG, Erlangen). Preoperative screw planning was then carried out on this data set in accordance with clinical standards. A verification of the registration was performed before each level was instrumented. By doing so, we assured sufficient precision in K-wire placement.

**Fig. 2:**
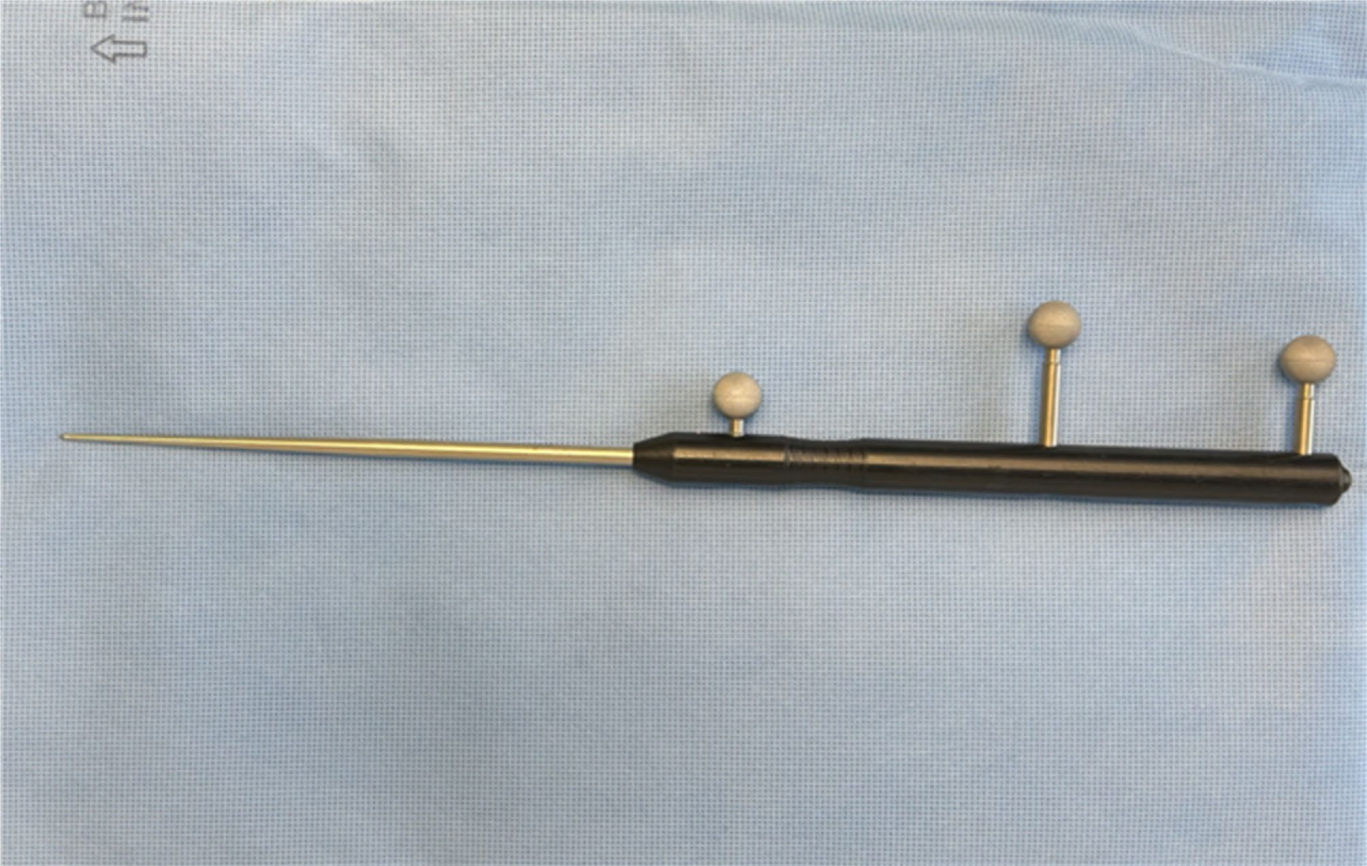
Navigation pointer.

**Fig. 3:**
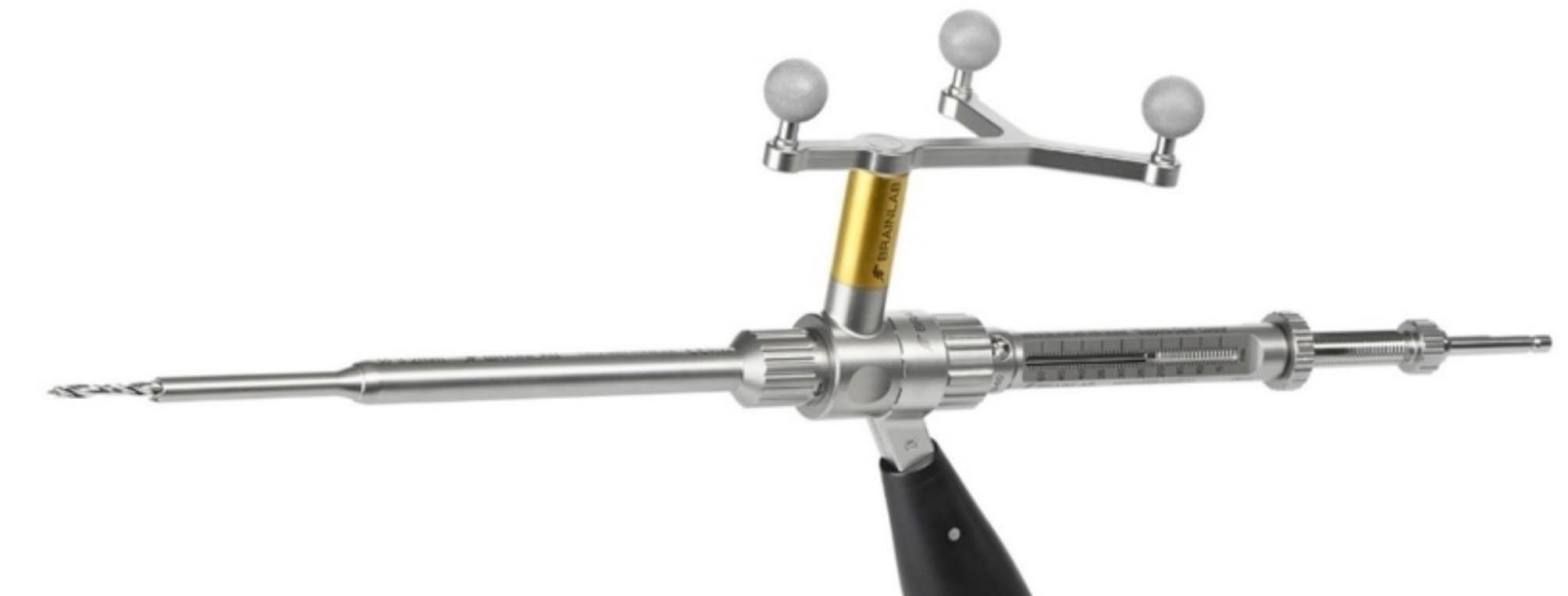
Hand-held drill guide.

In the group of conventionally navigated pedicle screws, the trajectories were located using the pointer tool (see Fig. 2). A 1.5 cm paramedian skin incision was performed over the entry point with sharp opening of the fascia and blunt dissection at the pedicle entry point. The corresponding hand-held drill guide (see Fig. 3) was then used to locate the entry point again. If the trajectory matched correctly, the guide was used to drill with the corresponding 2.6 mm drill bit and insert the respective 1.9 mm K-wire into the drill hole.

**Fig. 4:**
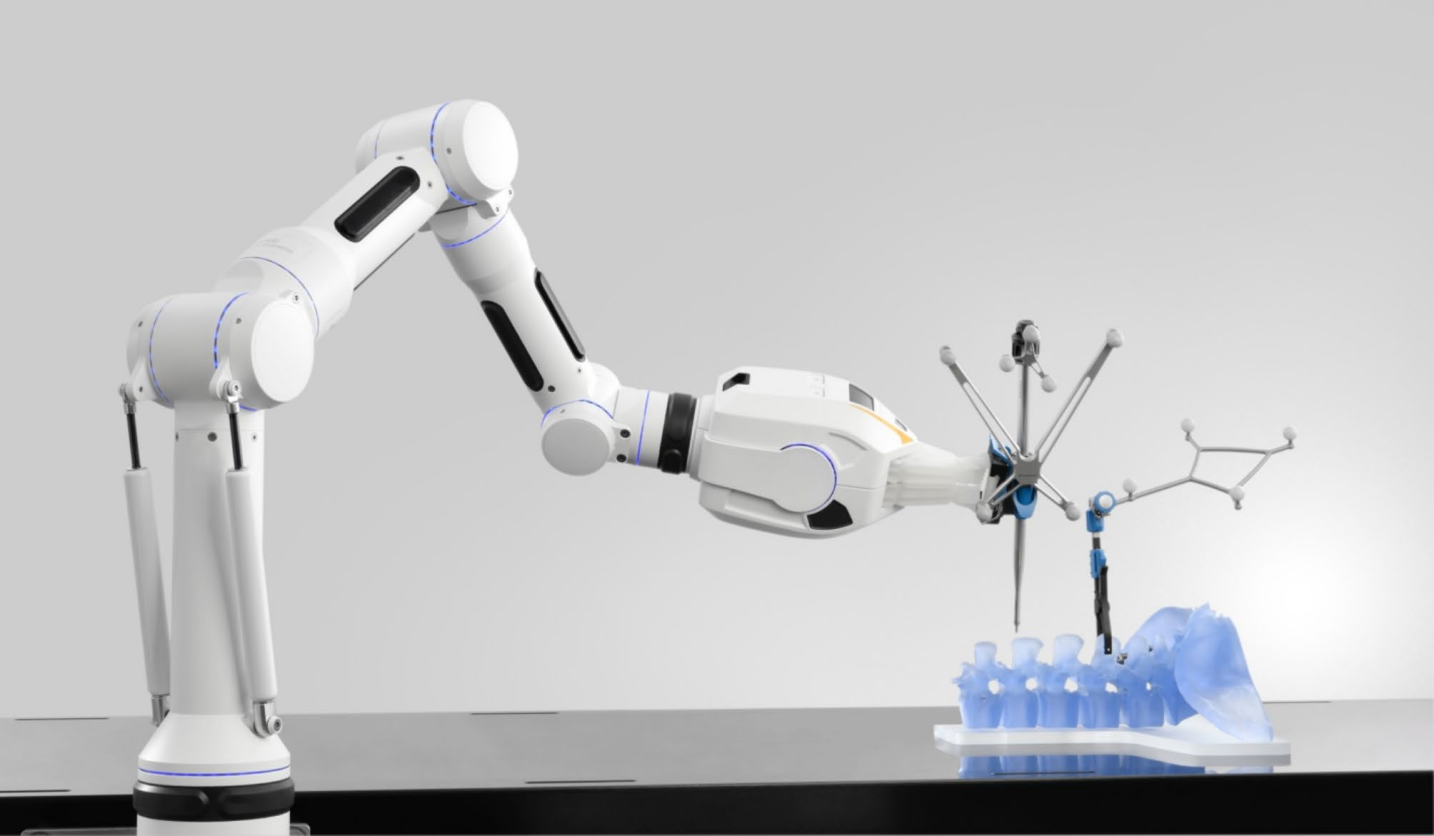
Robotic arm with drill guide.

**Fig. 5:**
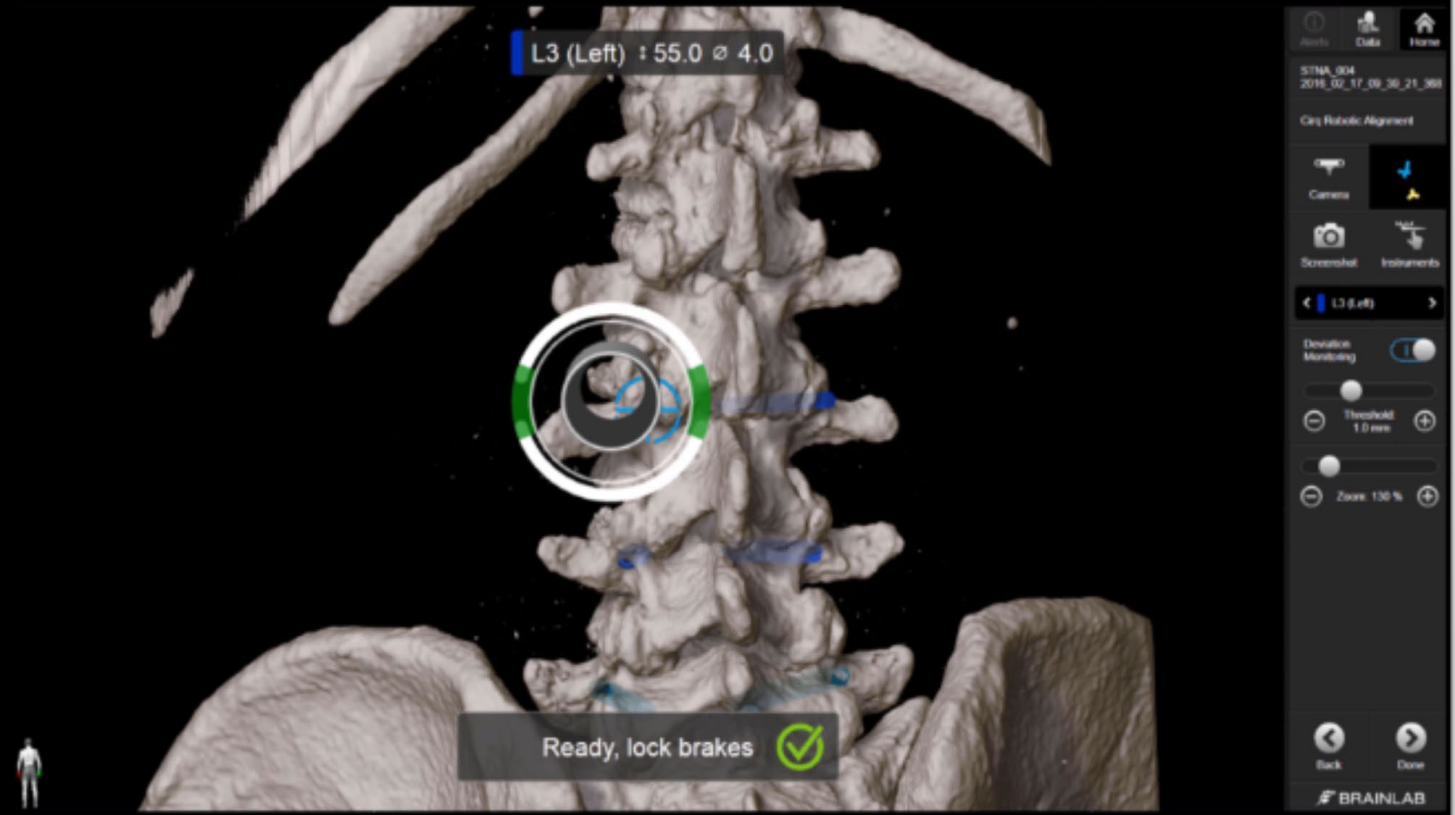
Positioning of the robotic arm close to the trajectory.

In the group of robot-assisted instrumentation, the trajectory for the skin incision was also located using the corresponding pointer. A 1.5 cm paramedian skin incision on the opposite side was performed over the entry point with sharp opening of the fascia and blunt dissection at the pedicle entry point. The robotic arm was then guided close to the planned trajectory (see Figs. 4 and 5) and the fine adjustment to control the trajectory was performed by the robotic system. After correct adjustment, the robotized sleeve was used to drill the 2.6 mm hole with the appropriate drill bit and insert the respective 1.9 mm K-wire into the drill hole.

On the first torso, conventional navigation was used on the left side, on the second torso on the right side. After finishing both sides, a 3D scan was carried out for radiological evolution.

**Fig. 6:**
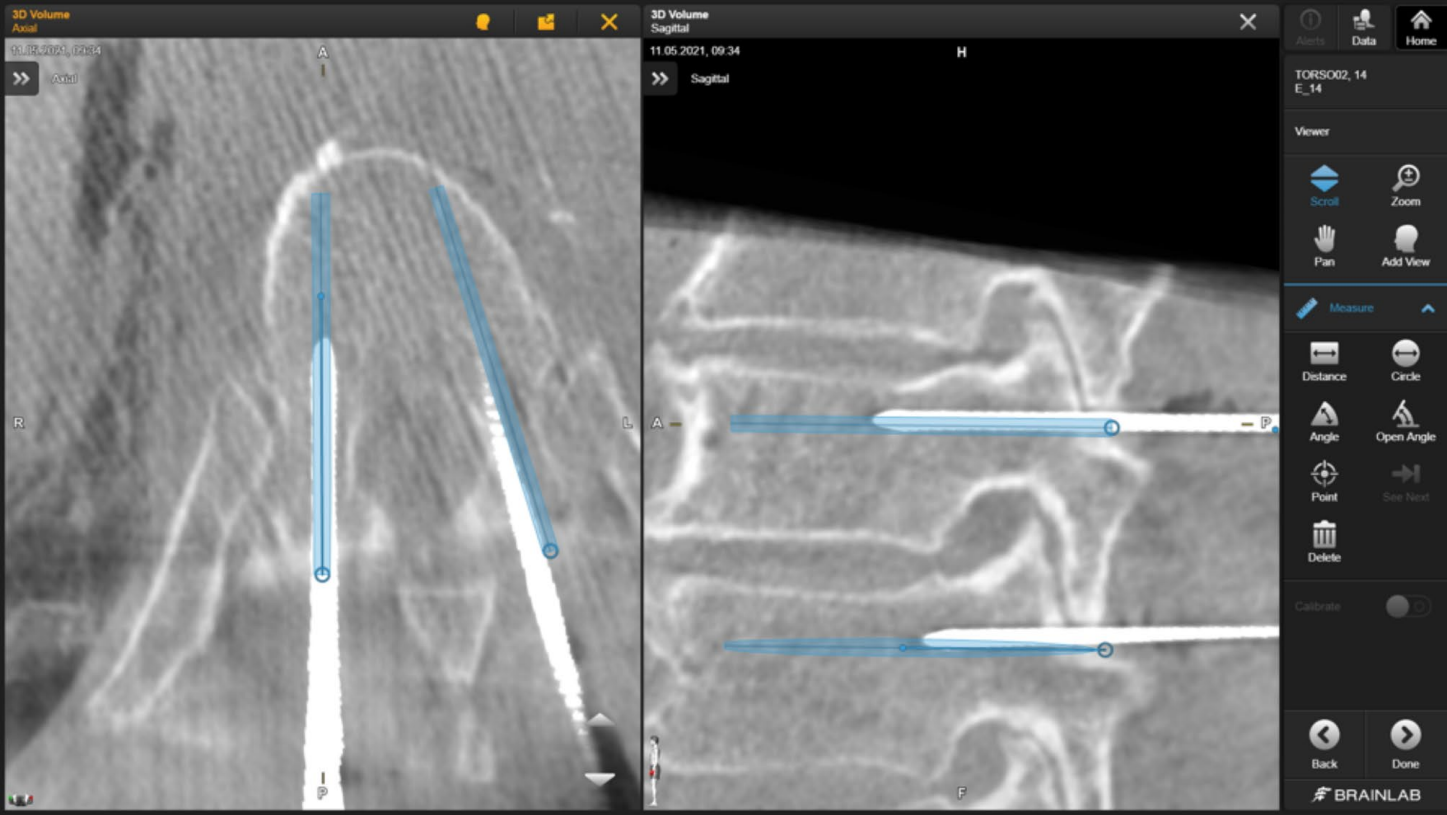
Image fusion (blue: planned trajectories; white: definitive K-wire position).

The navigation application which was used offers the feature of automated image fusion. The preoperative (planning) 3D scan containing the planned trajectories and the control 3D scan containing the definitive wire positions were merged into a single data set so that the trajectories previously planned as described above could be compared with the final wire position (see Fig. 6). First, the correct fusion was checked manually. Subsequently, the measurement tools available in the navigation application were used to measure the parameters listed below for any deviations using the dual control principle.

**Fig. 7:**
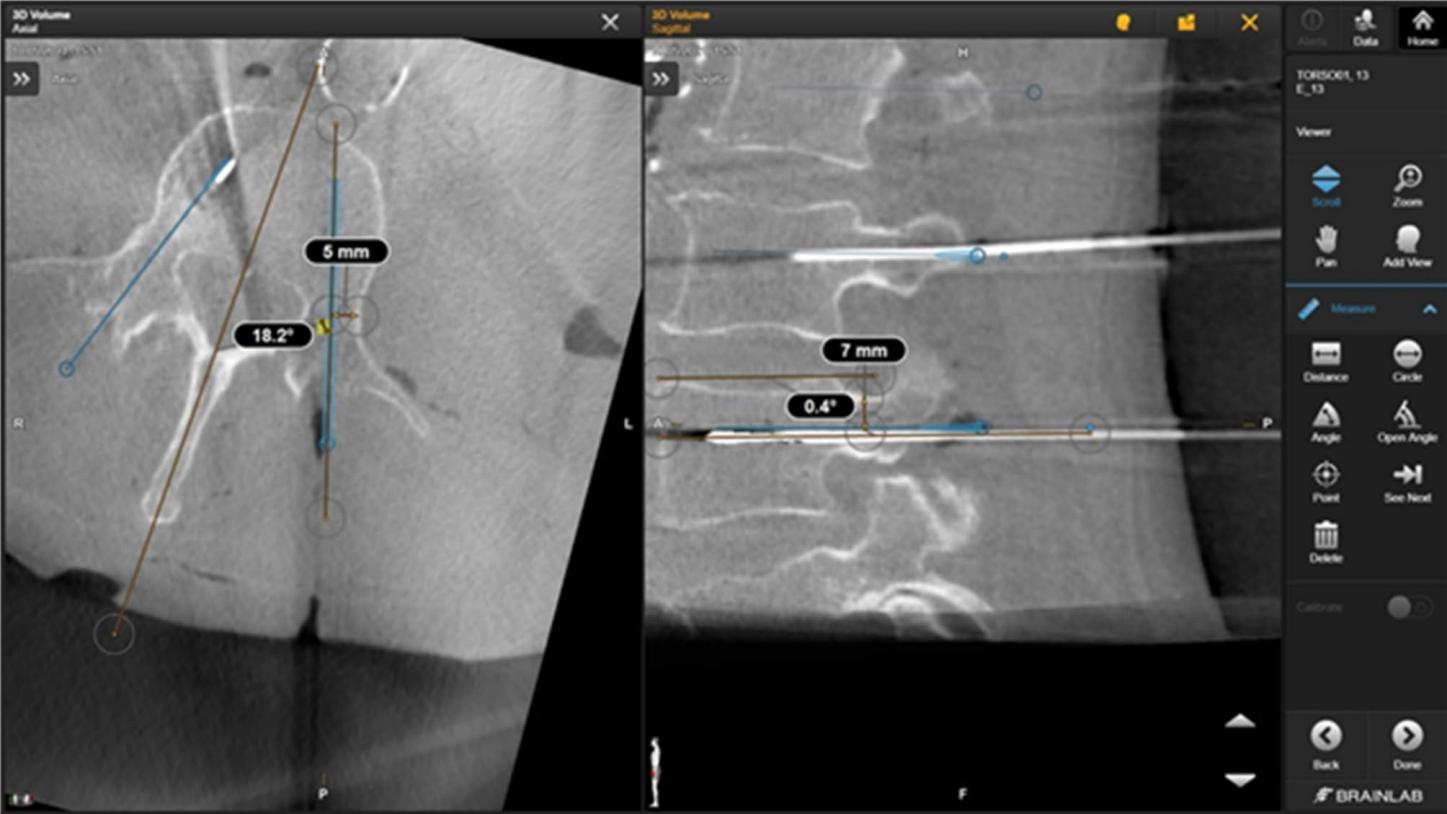
Measurements after image fusion.

The following parameters were used for evaluation:

1. Time from beginning of finding the trajectory to completion of wire positioning.
2. Distance of the wire to the planned trajectory in the pedicle in sagittal and axial projection (see Fig. 7).
3. Deviation of the angle of the wire to the planned trajectory in sagittal and axial projection.
4. Each subject was given a standardized questionnaire on subjective aspects of the system.
  - Clinical experience (years and spinal procedures performed).
  - Number of spinal procedures performed.
  - Subjectively required attention (1–5, 1 = low attention, 5 = high attention).
  - Reliability in positioning of implants and of the application (1–5, 1 = low safety, 5 = high safety).
  - Trust in the system (1–5, 1 = low trust, 5 = high trust).
  - Simplicity of application (1–5, 1 = not simple, 5 = very simple).
  - Possibility to dynamically change the procedure (1–5, 1 = no change possible, 5 = change possible at any time).
  - Recommendation (1–5, 1 = no recommendation, 5 = full recommendation).
  - Freedom of action (1–5, 1 = low freedom of action, 5 = high freedom of action).
  - Freetext/note.

In addition, the wire position was assessed corresponding to the Gertzbein and Robbins classifications. Transpedicular screw position is graded from A to E based on the extent by which the screw breaches the cortex of the pedicle:

- A: fully intrapedicular position without breach of the pedicle cortex.
- B: exceeding the pedicle cortex < 2 mm.
- C: exceeding the pedicle cortex 2–4 mm.
- D: exceeding the pedicle cortex 4–6 mm.
- E: exceeding the pedicle cortex > 6 mm or is outside of the pedicle.

Grade A and B can be considered as a satisfactory operation result. In grade C to E, neurological symptoms may occur and can be evaluated as an unsatisfactory surgical result^10^.

The statistical evaluation of the measurements was carried out in MS Excel (Microsoft Corporation, Redmond, USA).

## Results

### Precision

For the precision analysis, 89 of the 90 wires were available, since during the 3D scan one of the applied wires was dislocated and thus it was no longer evaluable in the control scan.

**Table 1:** Gertzbein/Robbins classification related to the K-wires.

Gertzbein and Robbins classification	Number of K-wires robot	Number of K-wires navigation
A	38	43
B	0	0
C	3	0
D	3	2
E	0	0

A total of 14% (6) of the wires set by the robot needed revision (defined as Gertzbein/Robbins C-E): 5 on the first torso and 1 on the second torso. 67% (4) of them were located on the cervical spine, (33%) 2 on the middle and lower thoracic spine. 4% (2) of the wires set by conventional navigation needed revision due to Gertzbein/Robbins C-E, both on the first torso, one located on the mid thoracic spine, one on the upper lumbar spine (see Table 1). The difference in the number of wires to be revised between robot and navigation was not significant for the first (*p* = 0.11) or the second torso (*p* = 0.16).

The measured deviation of the wire from the trajectory of the robot on the first torso was 1.1 mm (min 0 mm; max 7 mm; SD ± 1.5 mm), the angular deviation 1.2° (min. 0°; max. 7°; SD ± 1.7°) (see Table 2).

On the second torso, the deviation was on average 0.9 mm (min. 0 mm; max. 5 mm; SD ± 1.1 mm), the angular deviation 0.8° (min. 0°; max. 4°; SD ± 1.0°) (see Table 3). The difference in distance and angle deviation was not significant between the individual torsos (distance: *p* = 0.12, angle *p* = 0.07).

The deviation of the wire during navigation was 1.1 mm (min. 0 mm; max. 5 mm; SD ± 1.3 mm), the angular deviation was on average 1.5° (min. 0°, max. 6°; SD ± 1.6°) (see Table 2).

On the second torso, the deviation was on average 1.0 mm (min. 0 mm; max. 6 mm; SD ± 1.4 mm), the angular deviation analogous to the first torso 1.5° (min. 0°; max. 7°; SD ± 1.6°) (see Table 3). The difference in distance and angle deviation was not significant between the individual torsos/days (distance: *p* = 0.37, angle *p* = 0.45).

The comparison between the robot arm and conventional navigation on the first torso didn’t show a significant difference (distance: *p* = 0.39, angle: *p* = 0.24).

On the second torso, the difference in the distance was again not significant (*p* = 0.29), in the angular deviation the robot was significantly more precise (*p* = 0.005).

Averaged over both torsos, the following values were obtained:

The average distance deviation of the robot was 1.0 mm (min. 0 mm; max. 7 mm; SD ± 1.3 mm). The angular deviation was 1.0° (min. 0°; max. 7°; SD ± 1.4°) (see Table 4).

The deviation in conventional navigation was 1.0 mm (min. 0 mm; max. 6 mm; SD ± 1.3 mm), the angular deviation 1.5° (min. 0 mm; max. 7 mm; SD ± 1.6°) (see Table 4). The distance deviation was the same on average for both methods, there was no significant difference (*p* = 0.44). The difference in angular deviation was significant (*p* = 0.01).

**Table 2:** Precision analysis torso 1.

Torso 1	Average	Min.	Max.	SD
Distance deviation navigation	1.1 mm	0 mm	5 mm	± 1.3 mm
Distance deviation robot	1.1 mm	0 mm	7 mm	± 1.5 mm
Angular deviation navigation	1.5°	0°	5°	± 1.6°
Angular deviation robot	1.2°	0°	7°	± 1.7°

**Table 3:** Precision analysis torso 2.

Torso 2	Average	Min.	Max.	SD
Distance deviation navigation	1.0 mm	0 mm	6 mm	± 1.4 mm
Distance deviation robot	0.9 mm	0 mm	5 mm	± 1.1 mm
Angular deviation navigation	1.5°	0°	7°	± 1.6°
Angular deviation robot	0.8°	0°	4°	± 1.1°

**Table 4:** Total precision analysis.

Total	Average	Min.	Max.	SD
Distance deviation navigation	1.0 mm	0 mm	6 mm	± 1.3 mm
Distance deviation robot	1.0 mm	0 mm	7 mm	± 1.3 mm
Angular deviation navigation	1.5°	0°	7°	± 1.6°
Angular deviation robot	1.0°	0°	7°	± 1.4°

A significant difference in precision between the individual test subjects could not be shown in an ANOVA analysis (Analysis of Variance) between the test subjects for either the hand-guided navigation or the robot arm (distance deviation robot *p* = 0.3; distance deviation navigation *p* = 0.4; angle deviation robot *p* = 0.5; angle deviation robot *p* = 0.9).

The clinical experience in years and the number of spinal operations performed showed a clear correlation among the test subjects (correlation coefficient 1.0). The clinical experience in years was used for the correlation analyses.

A clear correlation between the experience of the test subjects and the individual precision could not be shown (see Table 5).

**Table 5:** Correlation between precision and experience.

	Correlation coefficient
Distance deviation navigation	-0.36
Distance deviation robot	0.01
Angular deviation navigation	0.13
Angular deviation robot	0.58

## Time

**Fig. 8:**
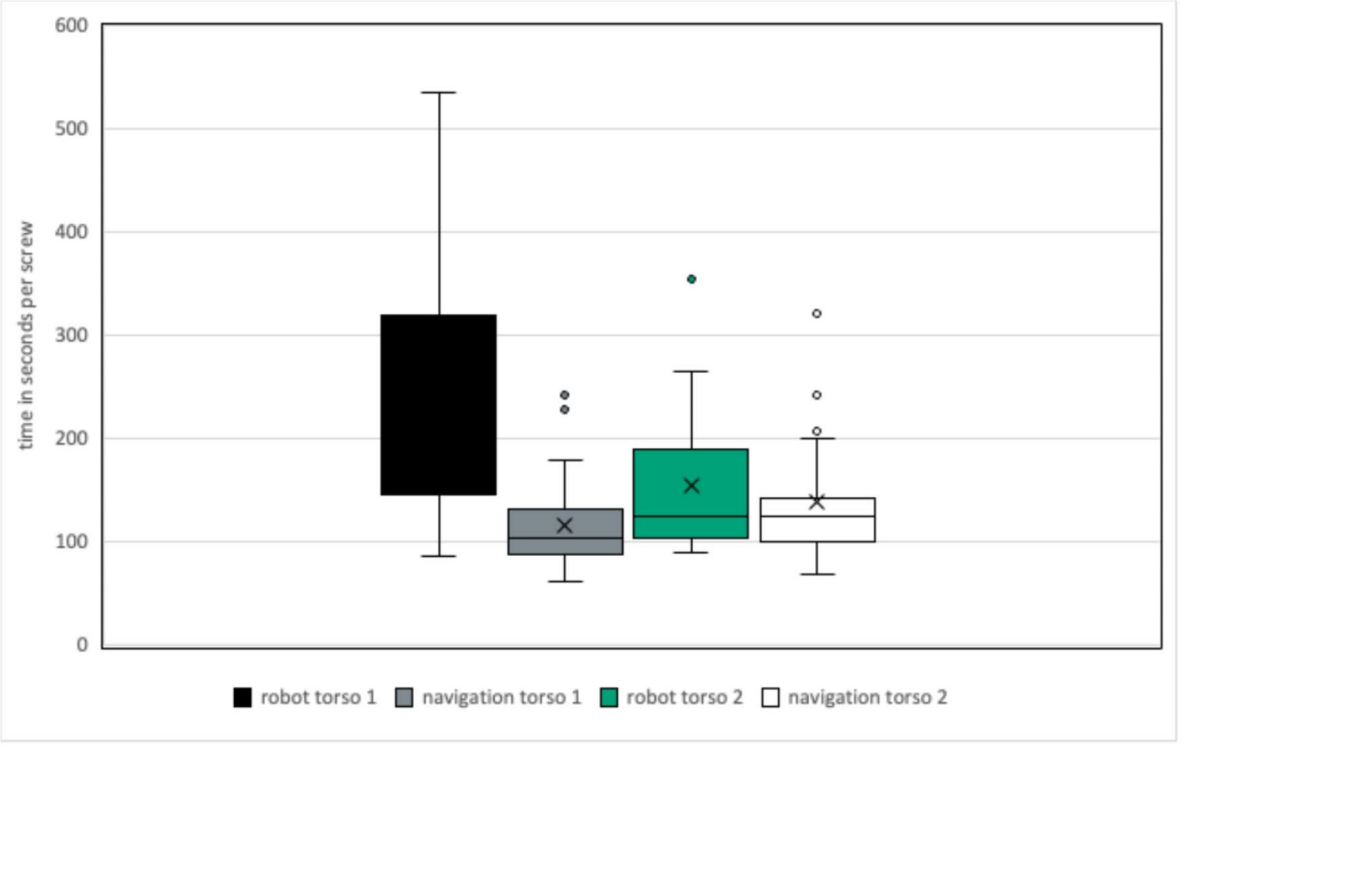
Statistical distribution of times per wire.

The time measurement showed a significantly longer duration per wire on the first torso of an average of 120s for the robot arm (236s (min. 86s; max. 536s; SD ± 131s) vs. 116s (min. 62s; max. 242s; SD ± 47s), *p* < 0.001). The second torso showed a not significant average of 16 s longer time per wire in the robotically assisted procedure (154s (min. 89s; max .355s; SD ± 68s) vs. 138s (min. 68s; max. 322s; SD ± 60s), *p* = 0.2) (see Tables 6 and 7; Fig. 8).

The time difference per wire in navigation was not significant across both torsos (*p* = 0.09).

For the vertebral bodies instrumented with the robot arm, there was a significant reduction in the mean duration of positioning per wire by 81s comparing torso 1 and torso 2 (*p* = 0.007).

**Table 6:** Time per wire on first torso.

Torso 1	Average	Min.	Max.	SD
Time per wire navigation	116s	62s	242s	± 47s
Time per wire robot	236s	86s	536s	± 131s

**Table 7:** Time per wire on second torso.

Torso 2	Average	Min.	Max.	SD
Time per wire navigation	138s	68s	322s	± 60s
Time per wire robot	154s	89s	355s	± 68s

In an ANOVA analysis, no significant difference in time per wire was found between the test subjects in either group (navigation or robot) (navigation *p* = 0.3, robot *p* = 0.06). As described above, clinical experience in years was used for the correlation analyses.

In the hand-guided navigation group, there was an inverse correlation between clinical experience and the average time per wire with a correlation coefficient of -0.97.

In the group of robot-assisted wire placement, this correlation could not be verified (correlation coefficient − 0.37).

## Subjective impression

The handling was consistently rated as good by all test persons. In the freetext the lack of possibility to deviate from the planned trajectory in the robotarm was criticized. There was no significant difference in the subjective impressions comparing navigation and robot except the possibility to change the procedure, here could be found a significantly more positive evaluation in favor of hand-guided navigation (*p* = 0.04) (see Tables 8 and 9).

**Table 8:** Subjective impression navigation.

Navigation	Average	Min.	Max.	SD
Subjectively required attention	4	3	5	1.2
Safety in the positioning	3.5	2	4	1.0
Trust in the system	4	3	5	1.2
Simplicity of application	4	4	4	0.0
Possibility to change the procedure	4.5	3	5	1.0
Recommendation	4.5	3	5	1.0
Freedom of action	4.5	4	5	0.6

**Table 9:** Subjective impression robot.

Robot	Average	Min.	Max.	SD
Subjectively required attention	4	3	5	1.2
Safety in the positioning	3.75	3	4	0.5
Trust in the system	3.5	3	5	1.0
Simplicity of application	4.25	4	5	0.5
Possibility to change the procedure	2.75	1	4	1.3
Recommendation	4.5	3	5	1.0
Freedom of action	4.25	3	5	1.0

## Discussion

A statistically significant difference in the precision between positioning with the robotic arm and conventional navigation could not be shown.

What is noticeable, though, in the data is the higher rate of wire misplacement (Gertzbein/Robbins C-E) of the robot compared to manual navigation, especially on the first torso. The above wire malpositions were mainly located in the cervical spine. Since decapitated human specimens were used in this study, the trajectories did not correspond to the usual clinical trajectories and were partially parallel to the table. It was not always possible to approach the wire positions correctly due to the larger structure of the front of the robotic arm. On the second torso, this was optimized by a correspondingly improved positioning of the torso, which was reflected by an improved wire position on the upper cervical spine. To achieve adequate precision, correct positioning and preoperative preparation must also be ensured for the robot.

The precision of both methods seems suitable for clinical use. The precision of the Cirq in our study on the second torso corresponds to the above optimizations with the systems of other manufacturers^11^. Nevertheless, the transfer of the cadaver model to a clinical setting must be interpreted with caution.

A time advantage using the robotic arm could not be demonstrated in the present study, which is in line with the current study situation. For example, a meta-analysis by Zhang et al. from 2019 showed comparable precision of different robotic systems in spinal surgery with simultaneously prolonged surgery^12^. However, the significant improvement in time per wire with the robotic arm on the second torso leads to several hypotheses. Firstly, it is to be expected that the observed longer duration per wire levels out due to an awaited steep learning curve. This hypothesis is supported by the work of Pojskic et al. who were also able to show a clear learning curve with the Cirq (here in clinical use)^13^. In their analysis of the Cirq, Gabrovsky et al. were able to demonstrate a significant correlation between the experience of the respective surgeon with the robotic arm and the time per wire and screw^14^.

Chesney et al. were also able to demonstrate this significant learning curve in a series of 714 screws placed with the Cirq^15^.

It could also be shown that an initial prolongation of the surgery when using a new and (for the surgeon) unknown tool can be observed regardless of the clinical experience of the respective surgeon.

The subjective survey of the participants also showed that a free hand during the procedure was rated as positive. Conversely, however, it was also criticized that the design of the robot’s “assisting hand” offers few opportunities for manual manipulation and thus for dynamic changes to the surgical plan. This should be possible with any technical aid in the operation room and should be mentioned here as a problem of the robot-assisted procedure.

The high purchase price and running costs of a robotic solution compared to navigation also need to be discussed. While the use of navigation and robot can be mapped via a separate OPS code in Germany (5-988.3 and 5-987), reimbursement of both operations by health insurance funds is currently pending. This means that the costs of the robotic arm are amortized more slowly compared to navigation due to its higher acquisition costs. Due to the comparable precision of navigation and robots, similar revision rates can be expected. Further studies are needed to investigate a long-term time advantage after completion of the learning curve and thus to examine the extent to which a reduction in operating time results in cost savings.

### Limitations

The study was conducted on decapitated human specimens. This showed an altered alignment around the remaining cervical spine as well as an altered anatomy with abnormal trajectories. For example, as described above, some trajectories were almost parallel to the operating table, which does not correspond to the usual clinical picture. Accordingly, the times and precision at these altitudes must be critically assessed. The study was conducted in a realistic setting. Nevertheless, not all aspects of a real surgery that could have an influence on the result (surgical drapes, surgical staff, structural conditions) can be represented here. The wire position does not always correlate exactly with the final screw position, screws can shift during final insertion, for example in osteoporotic or soft tissues. Further clinical studies with a larger number of inserted K-wires and even pedicle screws are necessary here.

## Conclusion

There were no differences between the test subjects in terms of time and precision. Existing navigation and robotic solutions are therefore also suitable for young residents and can and should be an integral part of clinical training. Nevertheless, it is important to learn and master the instruments in order to recognize technical problems and, if the technology fails, to be able to perform the procedure safely and quickly without risk to the patient^11^. Regarding minimally invasive spine surgery, the fluoroscopic technique of pedicle screw placement with a Jamshidi needle is also the standard for kyphoplasty, which continues to be of great importance in spinal surgery and is one of the basics for every spinal surgeon^11^.

Robotic- and navigation-assisted spine surgery show promising results and great potential for further investigations and they seem to be a safe and beneficial method for accurate pedicle screw placement. Nevertheless, further clinical studies with a higher number of cases are necessary to further evaluate the above results, especially the time and learning curve required and the precision in everyday clinical practice.

## Data Availability

The data that support the findings of this study are available upon reasonable request.
